# Differentiation of human induced pluripotent stem cells into erythroid cells

**DOI:** 10.1186/s13287-020-01998-9

**Published:** 2020-11-16

**Authors:** Mohsen Ebrahimi, Mehdi Forouzesh, Setareh Raoufi, Mohammad Ramazii, Farhoodeh Ghaedrahmati, Maryam Farzaneh

**Affiliations:** 1 Neonatal and Children’s Health Research Center, Golestan University of Medical Sciences, Gorgan, Iran; 2 Legal Medicine Organization of Iran, Legal Medicine Research Center, Legal Medicine organization, Tehran, Iran; 3 Faculty of Medical Sciences and Technologies, Science and Research Branch, Islamic Azad University, Tehran, Iran; 4 Kerman University of Medical Sciences, University of Kerman, Kerman, Iran; 5 Department of Immunology, School of Medicine, Isfahan University of Medical Sciences, Isfahan, Iran; 6 Physiology Research Center, Ahvaz Jundishapur University of Medical Sciences, Ahvaz, Iran

**Keywords:** Induced pluripotent stem cells, Erythrocytes, Reprogramming, Differentiation, Large-scale, Blood disorders

## Abstract

During the last years, several strategies have been made to obtain mature erythrocytes or red blood cells (RBC) from the bone marrow or umbilical cord blood (UCB). However, UCB-derived hematopoietic stem cells (HSC) are a limited source and in vitro large-scale expansion of RBC from HSC remains problematic. One promising alternative can be human pluripotent stem cells (PSCs) that provide an unlimited source of cells. Human PSCs, including embryonic stem cells (ESCs) and induced pluripotent stem cells (iPSCs), are self-renewing progenitors that can be differentiated to lineages of ectoderm, mesoderm, and endoderm. Several previous studies have revealed that human ESCs can differentiate into functional oxygen-carrying erythrocytes; however, the ex vivo expansion of human ESC-derived RBC is subjected to ethical concerns. Human iPSCs can be a suitable therapeutic choice for the in vitro/ex vivo manufacture of RBCs. Reprogramming of human somatic cells through the ectopic expression of the transcription factors (OCT4, SOX2, KLF4, c-MYC, LIN28, and NANOG) has provided a new avenue for disease modeling and regenerative medicine. Various techniques have been developed to generate enucleated RBCs from human iPSCs. The in vitro production of human iPSC-derived RBCs can be an alternative treatment option for patients with blood disorders. In this review, we focused on the generation of human iPSC-derived erythrocytes to present an overview of the current status and applications of this field.

## Introduction

Blood transfusion is the main therapeutic option and a crucial part of modern medicine for patients with severe anemia [1, 2]. A limited resource of blood, blood group compatibility (ABO and Rh antigens), and the risks of infection can present great challenges for blood transfusion [2, 3]. Therefore, any alternative solution methods would be most helpful for patients with rare blood groups [4]. Mature red blood cells (RBCs) or erythrocytes/erythroid cells in a complex process called erythropoiesis are produced from hematopoietic stem cells (HSCs) [5–7]. Erythroblasts (precursors of RBCs) are difficult to proliferate in vitro [2, 8, 9]. In past decades, several groups have generated erythrocytes from umbilical cord blood (UCB)-derived HSCs [2, 10]. Although multipotent HSCs have the capacity for self-renewal, the large-scale in vitro/ex vivo HSCs expansion and differentiation into RBCs is a difficult task [2, 11, 12]. Ex vivo cultured RBCs can also be obtained from immortalized erythroid precursors and pluripotent stem cells (PSCs) [13, 14]. Human PSCs including embryonic stem cells (ESCs) and induced pluripotent stem cells (iPSCs) have the potential to proliferate indefinitely in culture and give rise to lineages of ectoderm, mesoderm, and endoderm [15–17]. Therefore, much attention has focused on human PSCs to replace current transfusion banking [18, 19]. Several previous studies have revealed that human ESCs can differentiate into functional oxygen-carrying erythrocytes with normal function [18, 20, 21]. Unfortunately, the ex vivo expansion of human ESC-derived RBC is ethically and politically controversial [22, 23]. In contrast, human iPSCs have less ethical and social issues compared to human ESCs [24, 25]. Human iPSCs are produced by the manipulation of differentiated somatic cells [26–29]. Reprogramming of human somatic cells through the ectopic expression of transcription factors has provided a new avenue for disease modeling and regenerative medicine [16, 30]. As human iPSCs have similar properties with human ESCs, these cells can be a suitable therapeutic choice for the in vitro/ex vivo manufacture of RBCs to eliminate blood supply shortages [31, 32]. Various techniques have been developed to generate enucleated RBCs from human iPSCs [31, 33, 34]. Genome editing and human iPSCs technology has greatly accelerated the use of autologous transfusion therapies [35–38]. In this review, we focused on the generation of human iPSC-derived erythrocytes to present an overview of the current status and applications of this field.

### In vivo and in vitro erythropoiesis

**Fig. 1:**
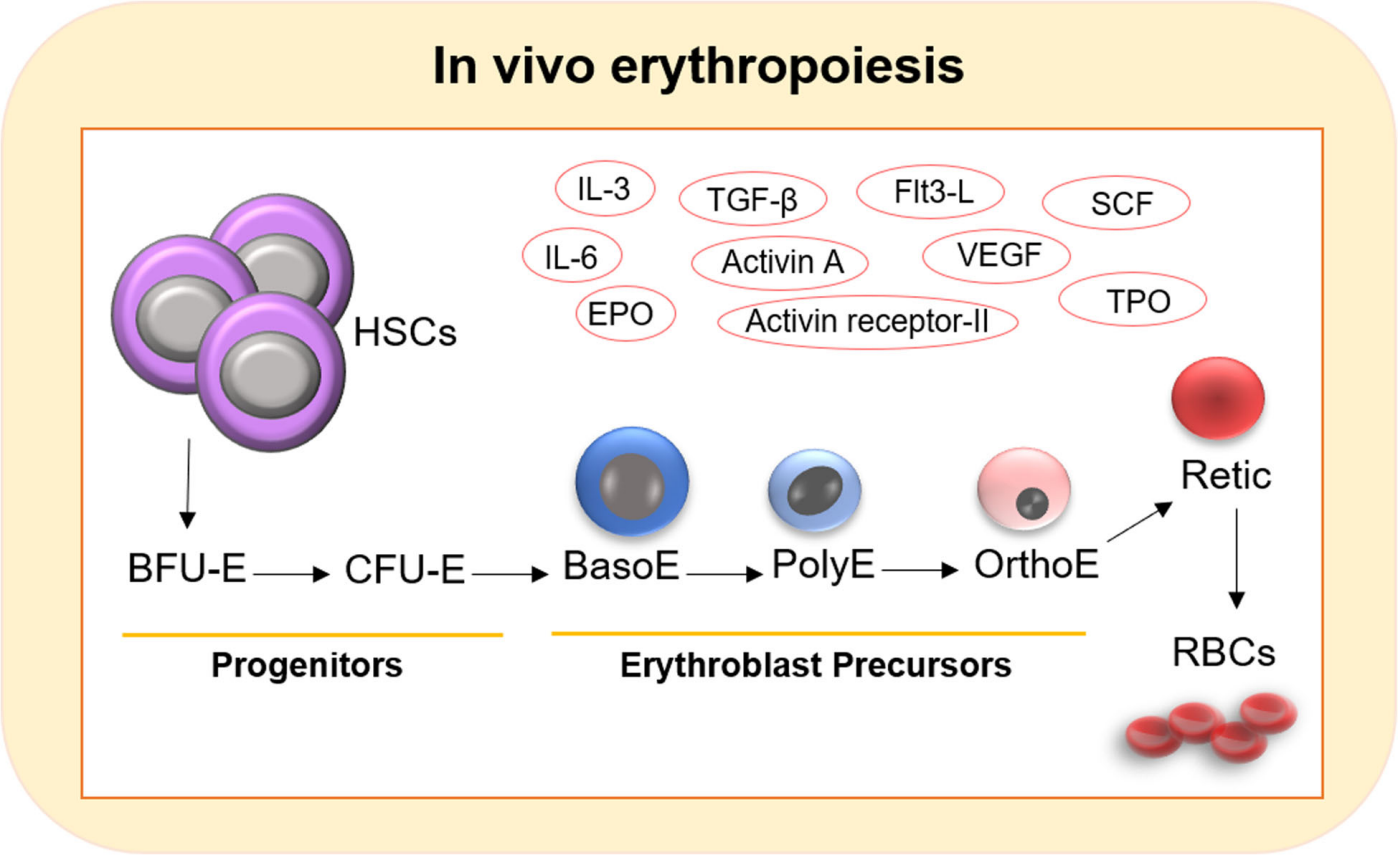
In vivo erythropoiesis. Erythropoiesis is a complex process in the bone marrow in which HSCs proliferate and give rise to erythroid committed progenitors (EPC) and mature red blood cells (RBCs). During development, HSCs lose their self-renewal properties and become restricted to generate burst-forming unit (BFU-E), colony-forming unit-erythroid (CFU-E), basophilic (BasoE), polychromatophilic (PolyE), orthochromatic erythroblasts (OrthoE), reticulocytes (Retic), and RBCs. Erythropoiesis is controlled by cell-cell/cell-matrix interactions along with several cytokines and growth factors including IL-3, IL-6, erythropoietin (EPO) (the main erythropoietic stimulating hormone), EPO-receptor, members of the transforming growth factor-β (TGF-β), activin A, activin receptor-II, Flt3 ligand (Flt3-L), vascular endothelial growth factor (VEGF), stem cell factor (SCF), and thrombopoietin (TPO)

Erythropoiesis is a complex process in the bone marrow in which HSCs proliferate and give rise to erythroid committed progenitors (EPC) and mature RBCs [39, 40]. Following differentiation toward the erythroid lineage, HSCs lose their self-renewal properties and become restricted to generate burst-forming unit (BFU-E), colony-forming unit-erythroid (CFU-E), basophilic (BasoE), polychromatophilic (PolyE), orthochromatic erythroblasts (OrthoE), reticulocytes (Retic), and RBCs [8] (Fig. 1). This developmental procedure is controlled by cell-cell/cell-matrix interactions along with several cytokines and growth factors including IL-3, IL-6, erythropoietin (EPO) (the main erythropoietic stimulating hormone), EPO-receptor, members of the transforming growth factor-β (TGF-β), activin A, activin receptor-II, Flt3 ligand (Flt3-L), vascular endothelial growth factor (VEGF), stem cell factor (SCF), thrombopoietin (TPO), and granulocyte colony-stimulating factor (G-CSF) [26, 41, 42]. Erythropoiesis is controlled and characterized via multiple transcriptional regulators, including myb, Sox6, Bcl11A, Gata1, and Klf1 [43, 44].

Up to date, several culture systems have been established to obtain a sufficient number of mature and functional RBCs in vitro [8]. Three culture systems including erythroid cells lines (the murine erythroleukemia cell line and the human K562 cell line), HSCs derived from peripheral blood (PB) and UCB, and stem cells (human ESCs, neonatal cord blood (CB), mesenchymal stem cells (MSCs), and human iPSCs) have been evaluated to obtain RBCs [26, 45]. An immortalized or continuous cell lines have a homogenous karyotype that can be altered following continuous cell culture, which might not be the most appropriate for pre-clinical studies or clinical applications [46]. In contrast, human PSCs-derived RBCs can be achieved in larger scale cultures [47, 48]. Many attempts have been made previously to establish all blood lineages, including lymphocytes, megakaryocytes, neutrophils, and RBCs from human ESCs [49–52]. Kaufman et al. for the first time revealed that human ESCs on the murine bone marrow cell line or a yolk sac endothelial cell line could give rise to erythroid cells [53]. Similar results were investigated by other lab using human fetal liver cells to improve the yield of human ESCs-derived erythroid cells [54]. However, the use of human ESCs has faced several challenges, including the host immune response toward ESCs and the ethical issues associated with the destruction of human embryos [55]. Differentiation of mouse iPSCs to blood cells has been investigated less intensively compared to the ESCs [56, 57]. Recently, human iPSCs have been studied as one of the potential sources for HSCs and RBCs [58–60].

### In vitro culture of human iPSC-derived RBCs

In 2006, murine iPSCs for the first time were obtained from somatic cells by using four transcription factors, Oct4/Klf4/Sox2/c-Myc [61]. In 2007, human iPSCs were generated from primary human fibroblasts using Oct4/Klf4/Sox2/LIN28 [62]. Human iPSCs enable researchers to generate PSCs using well-defined and highly reproducible protocols [63–65].

Although HSCs can be used for the treatment of hematological disorders [66, 67], the bone marrow biopsy is an invasive procedure with chronic graft-versus-host diseases (GVHD), morbidity, and mortality in patients who received allogeneic HSC therapy [68, 69]. Autologous HSCs are an alternative option with a lower mortality rate, though in some cases, genetic correction is necessary before autologous HSCs transplantation [70–72]. However, in vitro expansion of HSCs is one of the main hurdles of autologous HSCs [73, 74]. These limitations can be solved with a renewable source of cells. Human iPSCs as unlimited supplies of autologous cells can be an ideal candidate for genetic correction, differentiation to healthy HSCs, and autologous transplantation [67, 75].

**Fig. 2:**
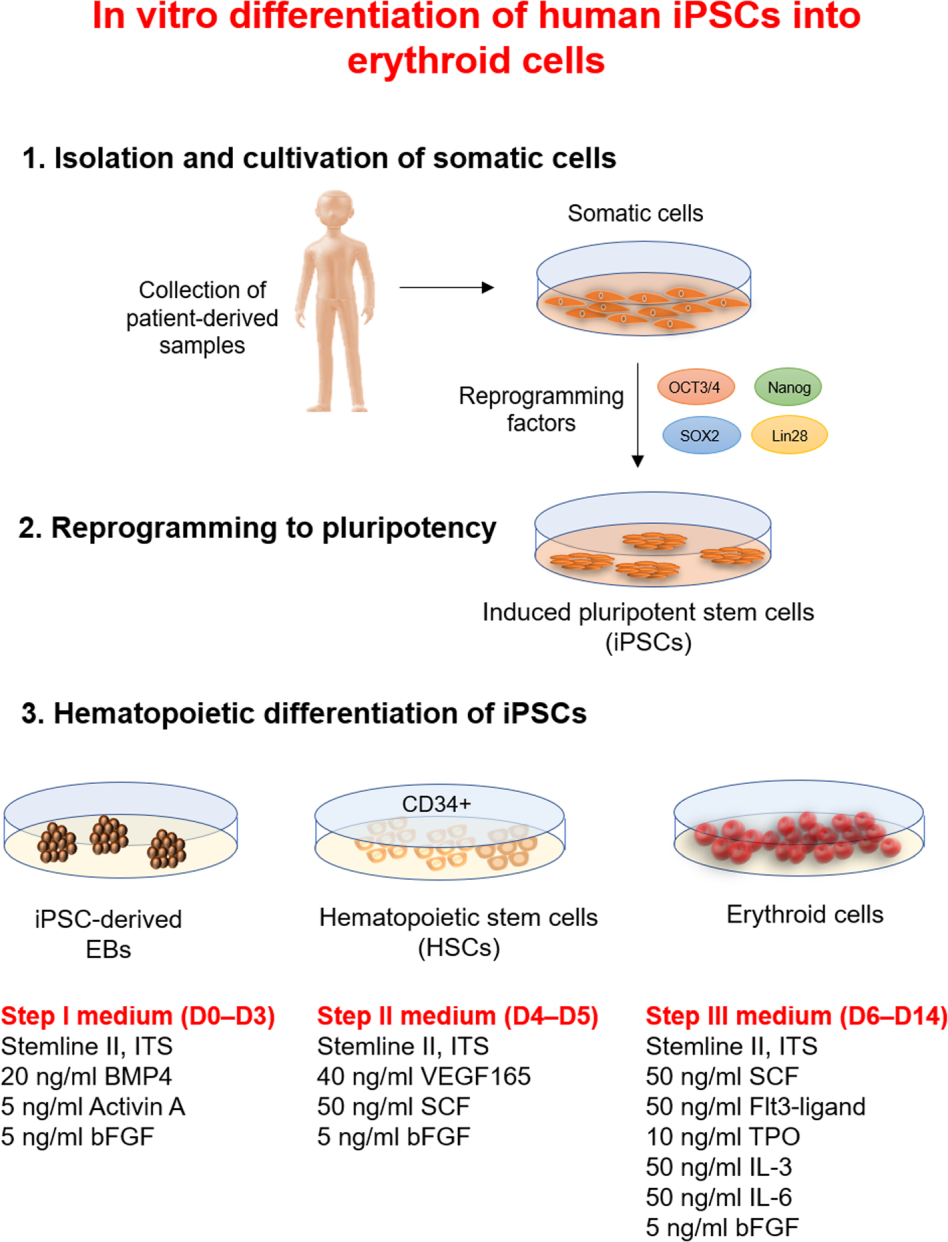
Differentiation of human iPSCs into RBCs. Human iPSCs can be produced from primary human fibroblasts using Oct4/Klf4/Sox2/LIN28. There are three steps for differentiation of iPSCs into RBCs, including the generation of human iPSCs, HSCs, and RBCs. Several growth factors and cytokines, including SCF, EPO, VEGF, IGF-1, ITS (insulin, transferrin, selenium), dexamethasone (glucocorticoid receptor agonist), TPO, FLT3, BMP4, IL-3, IL-6, and EPO have various functions on human iPSCs differentiation to the erythroid lineage

More recently, established iPSCs from human fibroblast cells represent a powerful tool for the investigation of early hematopoiesis [59, 76, 77]. One of the promising strategies for the use of iPSC is their capacity to differentiate into RBCs and to eliminate the allogeneic blood shortages [78, 79]. Two approaches including embryoid body (EB) formation (feeder-free culture) and co-culture of human iPSCs with feeder cells have been employed for the generation of HSCs from human iPSCs. Many studies have aimed to differentiate human iPSCs into RBCs using EB formation [31, 33, 34]. In general, there are three steps for differentiation of iPSCs into RBCs, including the generation of iPSCs, HSCs, and mature RBCs [33, 80] (Fig. 2). Many attempts have been made previously to achieve human iPSC-derived RBCs under conventional culture methods with SCF, EPO, VEGF, insulin-like growth factor I (IGF-1), dexamethasone (glucocorticoid receptor agonist), ITS (insulin, transferrin, and selenium), TPO, FLT3, BMP4, IL-3, IL-6, and EPO (Table 1). However, an ideal culture condition for human iPSC-derived RBCs should be able to generate large numbers of functional enucleated erythrocytes [31, 86]. Feeder cells as a major cellular component have been found to enhance hematopoiesis from human iPSCs [81, 82]. It has been shown that OP9 feeder cells as a mouse bone marrow stromal cell line may enhance the hematopoietic differentiation of human iPSCs [86]. Also, C3H10T1/2 feeder cells have the capacity to stimulate the hematopoietic differentiation of human iPSCs [38, 87]. Increasing in vitro evidence indicates that the cell type of origin and an epigenetic memory for iPSCs may influence on the hematopoietic differentiation of human iPSCs [34, 88, 89]. Compared with iPSC-derived fibroblast cells, the human CD34+ hematopoietic population with the features of MSCs might be more suitable for the hematopoietic differentiation of iPSCs [81]. Following differentiation, HSCs lose their repopulation capacity. Hence, CD34+ HPCs must be purified before starting the differentiation [38]. Human iPSC-derived CD43+ hematopoietic cells have a strongly glycosylated transmembrane sialomucin that can be a suitable option for in vitro erythropoiesis [79]. In HSCs, reactive oxygen species (ROS) can modulate a balance between proliferation and differentiation. In the early stage of hematopoietic differentiation, mitochondria and NADPH oxidases (NOX) are the main sources of ROS [90, 91]. NOX4 as the major NOX enzyme have been shown to play a significant role in the early stages of hematopoietic differentiation from iPSCs [85]. UM171 is a potent small molecule (HSC self-renewal agonist) that increases the derivation of HSPCs from human iPSCs in vitro [84, 92]. Choi et al. found some variations in the efficiency of human iPSCs differentiation into RBCs. While the pattern of hematopoietic differentiation was similar in seven tested lines [81], Dorn et al. reported that all human iPSCs could give rise to enucleated reticulocytes. But, the growth rate of erythroid cells from iPSC-derived CD34+ HSCs was slightly higher [34]. Uchida et al. demonstrated that compared to the yield of erythroid cells from PB erythroid progenitor-derived iPSCs, MSC-derived iPSCs produced more efficient definitive erythroid cells with higher b-globin expression [48]. Lapillonne et al. for the first time reported the complete differentiation of human iPSCs into definitive erythrocytes and RBCs with fetal hemoglobin [33]. Dias et al. revealed that the episomal reprogramming or transgene-free human iPSCs can be used for large-scale expansion of human iPSC-derived RBCs [82]. Olivier et al. illustrated the large-scale expansion of human iPSC-derived erythroid cells under feeder-free and serum-free culture conditions [83]. They used several small molecules such as StemRegenin (SR1, a dual RasGAP and ERK1/2 inhibitor), BIO (archetypal GSK3b inhibitor), CHIR99021 (GSK3b inhibitor), IBMX (nonspecific inhibitor of cAMP and cGMP phosphodiesterases), and A-A014418 (GSK3b inhibitor VIII) to promote erythroid differentiation of human iPSCs [83]. Recently, Bernecker et al. described a simplified cell culture system with low cytokine support (SCF, EPO, and IL-3) to generate prolonged human iPSC-derived RBCs [31]. Tursky et al. compared four serum and feeder-free iPSC hematopoietic differentiation protocols and investigated that two-dimensional (2D)-multistep protocol was simple and time- and cost-effective with the most efficient CD34+ progenitor cells [93].

**Table 1:** In vitro differentiation of human induced pluripotent stem cells (iPSCs) into red blood cells (RBCs)

Human iPSC cell source	Reprogramming transcription factors	Cluster of differentiation (CD) markers	iPSC culture condition	Results	Refs.
IMR90	POU5F1, SOX2, and NANOG	CD34+ and CD43+ (hematopoietic progenitors), CD31+ and CD43− (endothelial cells), CD43+, CD235a+, and CD41a+/− (erythro-megakaryopoietic)	α-MEM with 20% defined FBS, 100 ng/mL bFGF, OP9 feeder layer	Seven human iPSC lines could differentiate into RBCs with the similar pattern of differentiation	[81]
Fetal and newborn foreskin fibroblasts	POU5F1, SOX2, NANOG, and LIN28				
Adult skin fibroblasts	POU5F1, SOX2, and NANOG (M3-6) or POU5F1, SOX2, NANOG, and LIN28				
IMR90 and FD-136	pSin-EF2-Oct4-Pur, pSin-EF2-Sox2-Pur, pSin-EF2-Nanog-Pur and pSin-EF2-Lin28-Pur13	CD34 and/or CD45 (hematopoietic progenitors), CD36 and CD235a (erythroid cells), CD71 (transferrin receptor), CD45, CD34, and CD71 (hematopoietic and erythroid cells)	EB formation on a cellular stroma 100 ng/mL SCF, 100 ng/mL TPO, 100 ng/mL FL, 10 ng/mL BMP4, 5 ng/mL VEGF, 5 ng/mL IL-3, 5 ng/mL IL-6, 3 U/mL Epo, 10 μg/mL insulin, 3 U/mL heparin	The complete differentiation of human iPSCs into definitive erythrocytes and RBCs with fetal hemoglobin	[33]
Human adult and fetal fibroblasts	POU5F1, SOX2, and NANOG	CD235a+ and CD45− (leukocyte-free RBCs), CD34+ or CD31+ (erythroid cells)	100 ng/mL ZbFGF, OP9 feeder layer, serum free medium, SCF, G-CSF, GM-CSF, IL3, IL6	The episomal reprogramming or transgene-free human iPSCs for large-scale expansion of RBCs	[82]
Neonatal fibroblasts	Episomal vectors that express OCT4, SOX2, NANOG, LIN28, MYC, KLF4, and LT				
Human cord blood	OCT4 and SOX2 alone (CD34-2F-iPSC) or expressing OCT4, SOX2, KLF4, and c-MYC (CD34-4F-iPSC)	CD34+ (iPSCs), CD45+/CD34+ (HSCs), CD45+/CD34− (myeloid precursors), GPA+/CD45− (erythroid cells), CD36 and CD71 (primitive erythroid cells)	10% human plasma, 10 μg/mL insulin, 330 μg/mL human holotransferrin, 100 ng/mL SCF, 100 ng/mL TPO, 100 ng/mL Flt3-L, 5 ng/mL IL-3, 5 ng/mL IL-6, 5 ng/mL VEGF, 10–20 ng/mL BMP4, 3 U/mL EPO	The growth rate of erythroid cells from iPSC-derived CD34+ HSCs was slightly higher	[34]
iPSC line (33D6), iPSC lines from fibroblast cells (blood group O RhD2), and peripheral blood		CD144+/CD31+ (endothelial cells), CD31, CD34, CD36, CD41a, CD43, CD44, CD45, CD71, and CD235a	Stemline II medium, 20 ng/mL bFGF, 20 ng/mL recombinant vitronectin, 1 mM StemRegenin (SR1), 1 mM hydrocortisone, 30–50 ng/mL SCF, 16.7 ng/mL Flt3-ligand, 10 ng/mL Wnt3A, 2 mM GSK3b inhibitor VIII or A-A014418, 6.7–20 ng/mL BMP4, 6.7 ng/mL IL-3, 6.7 ng/mL IL-11, 50 mM IBMX, 1.3 U/mL EPO, 30 ng/mL VEGF, 10 ng/mL FGFa, 10 ng/mL IGF, 10 ng/mL TPO, 5 mg/mL heparin, 50 mM IBMX, 0.4 ng/mL b-estradiol	The large-scale expansion of human iPSC-derived erythroid cells under feeder-free and serum-free culture condition	[83]
Cord blood CD34+ cells	OCT4, SOX2, KLF4, and c-Myc	CD43+ (hematopoietic progenitors), CD36, CD235a, CD45, CD71 (hematopoietic markers), CD31, CD144, CD41a, CD309, and CD4	VEGF, BMP4, Flt3-ligand, IL-3, IL-6, SCF, TPO, EPO	Human iPSC-derived CD43-expressing hematopoietic cells are a suitable option for in vitro erythropoiesis	[79]
PBMCs or MSCs from SCD patients	Oct4, Klf4, Sox2, and c-Myc	CD36+/CD71+ (peripheral blood erythroid progenitors (EP)), CD31, CD34, CD41a, CD43, CD45, CD71, CD73, CD144, CD235a, CD309	IDMEM medium, 0.2 mg/mL insulin, 0.11 mg/mL transferrin, 0.1 μg/mL sodium selenite, 0.45 mM a-mono-thioglycerol, 50 μg/mL AA, 20 ng/mL VEGF, 50 ng/mL SCF, 50 ng/ml fms-related tyrosine kinase 3 ligand, 50 ng/mL TPO, 5 μg/mL IL-3, 10 ng/mL BMP4, 5 U/ml EPO	MSC-derived iPSCs produced more efficient definitive erythroid cells with higher b-globin expression	[48]
Human urine	OCT4, SOX2, KLF4, and MYC	CD34, CD43, CD45, CD31, CD144, CD235a, CD11b, CD14, CD3, CD4, CD5, CD7, CD8a	Matrigel, mTeSR1 medium, stemline II, ITS, 20 ng/mL BMP4, 5 ng/mL Activin A, 5 ng/mL bFGF, 40 ng/mL VEGF, 50 ng/mL SCF, 50 ng/mL Flt3-ligand, 10 ng/mL TPO, 50 ng/mL IL-3, 50 ng/mL IL-6,	UM171 improved in vitro derivation of HSCs from human iPSCs	[84]
Cord blood CD34+ cells and CD36+ erythroblasts	OCT4, SOX2, KLF4, and c- MYC	CD34+/CD45+ (hematopoietic progenitors), CD36+/CD45+ (erythroid precursors)	Matrigel, STEMdiff™ APEL™2 medium, 5% PFHM-II Protein-free Hybridoma Medium, 5 ng/mL IL-3, 100 ng/mL SCF, 3 U/mL EPO, 10% human plasma, 10 μg/mL insulin, 330 μg/mL human holotransferrin	Prolonged human iPSC-derived RBCs in a simplified cell culture system with low cytokine support	[31]
WT-iPSC line		CD34, CD38, CD45, CD90, CD117, CD133	Vitronectin, OP9 feeder layer, MEM medium with 10% FBS, 100 μM MTG, 50 μg/mL AA	NOX4 has a significant role in the early stages of hematopoietic differentiation from iPSCs	[85]
Bone marrow stromal cells from a SCD patient		CD31, CD34, CD36, CD38, CD41a, CD43, CD45, CD45RA, CD49f, CD71, CD73, CD90, CD144, CD184	mTeSR1 media, Matrigel, IMDM, C3H10T1/2 feeder cells, 1% ITS, 50 mg/mL AA, 0.45 mM a-monothioglycerol, 20 ng/mL human VEGF, 15% FBS or 20% KSR, OP9 feeder cells, 50 ng/mL FL, 50 ng/mL TPO, 5 ng/mL IL3, 50 ng/mL SCF, 5 U/mL EPO, and 10 ng/mL BMP4, 1.0 μM estradiol, 1.0 μM dexamethasone, 2% BSA, 0.56 mg/mL transferrin	Serum-free iPSC sac-derived erythroid differentiation	[38]

*IMR90* human fetal lung fibroblasts, *PBMCs* peripheral blood mononuclear cells, *IDMEM* Iscove’s modified Dulbecco’s medium, *SCD* sickle cell disease, *FD-136* skin primary fibroblast cell line, *OP9* mouse bone marrow stromal cell line, *EB* embryoid body, *SCF* stem cell factor, *TPO* thrombopoietin, *FLT3* Fms-related tyrosine kinase 3 ligand, *FL* FLT3 ligand, *BMP4* bone morphogenetic protein 4, *VEGF* vascular endothelial growth factor, *IL-3* interleukin-3, *EPO* erythropoietin, *ZbFGF* zebrafish basic fibroblast growth factor, *HSCs* hematopoietic stem cells, *IGF* insulin-like growth factor, *IBMX* isobutyl methyl xanthine, *MTG* monothioglycerol, *AA* ascorbic acid, *KSR* knockout serum replacement, *BSA* bovine serum albumin, *ITS* insulin, transferrin, selenium

### Primary technical challenges for the clinical application of iPSC-derived RBCs

The in vitro production of human iPSC-derived RBCs can be an alternative treatment option for patients with blood disorders [94]. Many attempts have been examined to differentiate iPSCs into RBCs, but no clinical trials using iPSC-derived RBCs transfusion have been conducted [60]. Table 2 shows patient-specific iPSCs models of hematological disorders.

**Table 2:** Patient-specific iPSC models of hematological disorders

Authors	Disorder	iPSC cell source	Ref.
Ye et al. 2009	Myeloproliferative disorders (MPDs)	iPSCs from peripheral blood CD34+ cells of patients with MPDs	[95]
Zou et al. 2011	Chronic granulomatous disease (CGD)	iPSCs from patient with X-linked CGD	[96]
Kumano et al. 2012	Chronic myelogenous leukemia (CML)	iPSCs from imatinib-sensitive CML patient	[97]
Chang et al. 2012	α-Thalassemia (α-Thal)	iPSCs from α-Thal fibroblasts	[98]
Garçon et al. 2013	Diamond Blackfan anemia (DBA)	iPSCs from fibroblasts of DBA patient	[99]
Bedel et al. 2013	CML	iPSCs from CD34+ blood cells isolated from CML patients	[100]
Yuan et al. 2013	Paroxysmal nocturnal hemoglobinuria (PNH)	iPSCs from adult male dermal fibroblasts	[101]
Saliba et al. 2013	Polycythemia vera (PV)	iPSCs from 2 polycythemia vera patients carrying a heterozygous and a homozygous mutated JAK2 JAK2^V617F^	[102]
Sakurai et al. 2014	Familial platelet disorder (FPD)/AML	iPSCs from three distinct FPD/AML pedigrees	[103]
Sun et al. 2014	Sickle cell disease (SCD)	iPSCs from patient with SCD mutation	[104]
Ye et al. 2014	PV	iPSCs from PV patient blood	[105]
Xie et al. 2014	β-Thalassemia (β-Thal)	iPSCs from patient with β-Thal	[106]
Amabile et al. 2015	CML	Primary bone marrow cells obtained from a BCR-ABL-positive CML patient	[107]
Ge et al. 2015	DBA	iPSCs from DBA patients carrying RPS19 or RPL5 mutations	[108]
Park et al. 2015	Hemophilia A (HA)	iPSCs from patients with chromosomal inversions that involve introns 1 and 22 of the F8 gene	[109]
Kotini et al. 2015	Myelodysplastic syndromes (MDS)	iPSCs from hematopoietic cells of MDS patients	[110]
Huang et al. 2015	SCD	iPSCs from adult patients of SCD, which harbor the homozygous β^s^ mutation in the HBB gene	[111]
Chang et al. 2015	Severe combined immunodeficiency (SCID)	iPSCs from SCID patients with Janus family kinase (JAK3)-deficient cells	[112]
Menon et al. 2015	X-linked severe SCID (SCID-X1)	iPSCs from SCID-X1 patients	[113]
Ingrungruanglert et al. 2015	Wiskott-Aldrich syndrome (WAS)	iPSCs from patients with mutations in WASP	[114]
Wu et al. 2016	HA	iPSCs from peripheral blood from severe HA patients	[115]
Pang et al. 2016	HA	iPSCs from patients with severe HA	[116]
Niu et al. 2016	β-Thal	iPSCs from patient with β-Thal	[117]
Laskowski et al. 2016	WAS	iPSCs from CD34+ hematopoietic progenitor cells of a WAS patient	[118]
Doulatov et al. 2017	DBA	iPSCs from skin fibroblasts from DBA patient	[119]
He et al. 2017	Hemophilia B (HB)	iPSCs from HB patient	[120]
Chao et al. 2017	Acute myeloid leukemia (AML)	iPSCs from AML patient	[121]
Kotini et al. 2017	AML	iPSC from patients with low-risk MDS (refractory anemia [RA]), high-risk MDS (RA with excess blasts [RAEB]) and secondary AML (sAML or MDS/AML from preexisting MDS)	[122]
Miyauchi et al. 2018	CML	iPSCs from the bone marrow of two CML-CP patients	[123]
Olgasi et al. 2018	HA	iPSCs from peripheral blood (PB) CD34+ cells of HA patient	[124]
Ramaswamy et al. 2018	HB	iPSCs from HB patients	[125]
Lyu et al. 2018	HB	iPSC from peripheral blood mononuclear cells (PBMNCs)	[126]
Cai et al. 2018	β-Thal	iPSCs from patient with β-Thal	[127]
Wattanapanitch et al. 2018	HbE/β-Thal	iPSCs from Skin cells of HbE/β-Thal patients	[128]
Sfougataki et al. 2019	β-Thal, SCD, DBA, severe aplastic anemia (SAA), dedicator of cytokinesis 8 (DOCK8) immunodeficiency	iPSCs from human bone marrow-derived mesenchymal stromal cells (BM-MSCs)	[129]
Kohara et al. 2019	Type IV congenital dyserythropoietic anemia (CDA)	iPSCs from CDA patient carrying the KLF1 E325K mutation	[130]
Hoffmann et al. 2020	Severe congenital neutropenia (SCN)	iPSCs from a SCN patient with a nonsense mutation in the glucose-6-phosphatase catalytic subunit 3 (G6PC3) gene	[131]

Before iPSC-derived RBCs derivatives can be used in the clinic, it is essential to found the risks and process-related challenges associated with the generation of late-stage maturity RBCs in vitro [132, 133]. The technology of manufacturing functional erythroid cells from iPSCs needs a sufficient number of functional RBCs in a serum free-liquid culture system or chemically defined media, which is necessary for any potential clinical trials [134, 135]. Human iPSCs may be considered as an unlimited source of RBCs than HSCs, but generating mature RBCs from iPSCs is still an inefficient process and less strict experiment protocols using low-cost media and reagents are needed [136]. Thus, the challenge for large-scale expansion of iPSC-derived erythroid cells needs to be overcome [8, 60].

**Fig. 3:**
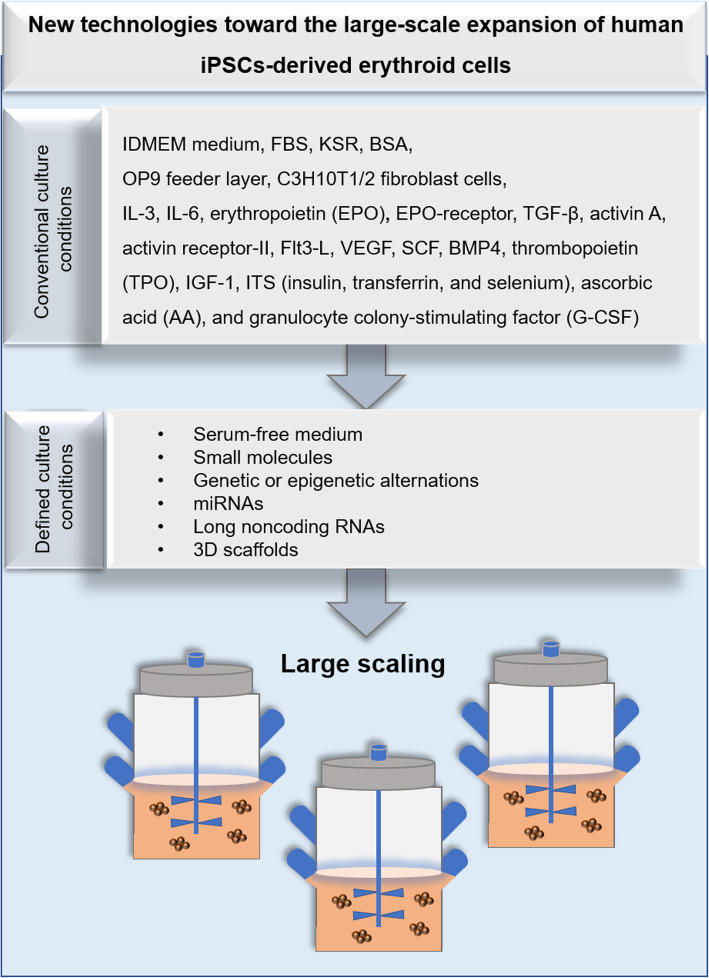
New technologies toward the large-scale expansion of human iPSC-derived erythroid cells. Conventional differentiation methods compared with the novel growth factor- and serum-free culture approaches for erythroid differentiation of human iPSCs

The use of small molecules as substitutes for growth factors or various cytokines can reduce side effects and media costs [137, 138]. Further studies are necessary to understand which genetic or epigenetic alternations improve the terminal differentiation of iPSC-derived erythroid cells [139]. Recent studies have shown that histone deacetylases such as histone deacetylase 2 (HDAC2) are the critical regulator for chromatin condensation in mouse erythroblasts [140]. Administration of HDAC2 inhibitors can suppress the terminal differentiation of human erythroid precursors [139]. It is therefore conceivable that HDAC2 activators may enhance chromatin condensation of iPSC-derived erythroid cells [141]. MicroRNAs are important regulators that downregulate the expression of target genes [142, 143] and improve the maintenance of immature hematopoietic cells and terminal erythroid differentiation [58, 143]. Therefore, different combinations of microRNAs may increase the numbers of iPSC-derived mature RBCs [139]. In addition to microRNAs, long noncoding RNAs (lncRNAs) have recently been reported that can determine the fate of stem cells [144]. A recent study has shown that long intergenic noncoding RNA erythroid prosurvival (lincRNA-EPS) can suppress apoptosis and facilitate erythropoiesis [145–147]. In this regard, lncRNAs might allow the generation of functional and mature RBCs from iPSCs [148, 149]. Several groups have recently shown that 3D scaffolds such as poly (D, L-lactide-co-glycolide), polyurethane, collagen type I, and porous polyvinyl fluoride resin can mimic the bone marrow niche and improve maintenance of immature hematopoietic cells [150–154]. Although the in vitro maturation of iPSC-derived RBCs still presents several barriers, the cultured erythroid cells from iPSCs provide an important step toward fully defined and animal-free cultivation protocols that can be applied for transfusion medicine [67]. Figure 3 shows new technologies toward the large-scale expansion of human iPSC-derived erythroid cells.

## Conclusion

Reprogramming of somatic cells to the pluripotent state has been suggested as an alternative source and a novel opportunity for patient-specific stem cell-based therapies, modeling of RBCs diseases, and drug testing [155]. Previous studies have shown that human iPSCs can give rise to erythroid cells, while in vitro derivation and maintenance of enucleated erythrocytes have still been challenging [86]. Also, many hurdles such as reprogramming without retroviruses, large scale and cost-effective production of iPSC-derived enucleated RBCs, and defined xenogenic-free conditions remain to be improved before human iPSC-based therapy [156, 157]. According to good manufacturing practice (GMP) guidelines, the establishment of iPSC-derived RBCs using a reproducible, defined, and simple method will ease the translation of iPSCs into the clinic [93, 158, 159].

## Data Availability

The datasets used and/or analyzed during the current study are available from the corresponding author on reasonable request.

## Abbreviations

AA: Ascorbic acid
BMP4: Bone morphogenetic protein 4
BSA: Bovine serum albumin
EB: Embryoid body
ESCs: Embryonic stem cells
EPO: Erythropoietin
FD-136: Skin primary fibroblast cell line
GVHD: Graft-versus-host diseases
FLT3: Fms-related tyrosine kinase 3 ligand
FL: FLT3 ligand
GMP: Good manufacturing practice
HDAC2: Histone deacetylase 2
HSCs: Hematopoietic stem cells
IBMX: Isobutyl methyl xanthine
IGF: Insulin-like growth factor
IDMEM: Iscove’s modified Dulbecco’s medium
IMR90: Human fetal lung fibroblasts
IL-3: Interleukin-3
ITS: Insulin, transferrin, selenium
iPSCs: Induced pluripotent stem cells
LncRNAs: Long noncoding RNAs
LincRNA-EPS: Long intergenic noncoding RNA erythroid prosurvival
KSR: Knockout serum replacement
MTG: Monothioglycerol
OP9: Mouse bone marrow stromal cell line
PBMCs: Peripheral blood mononuclear cells
PSCs: Human pluripotent stem cells
SCF: Stem cell factor
SCD: Sickle cell disease
RBC: Red blood cells
TPO: Thrombopoietin
UCB: Umbilical cord blood
VEGF: Vascular endothelial growth factor
ZbFGF: Zebrafish basic fibroblast growth factor
